# Evaluation of lablab bean [*Lablab purpureus* (L.) sweet] genotypes: unveiling superior pod yield, nutritional quality, and collar rot resistance

**DOI:** 10.3389/fnut.2023.1243923

**Published:** 2024-01-11

**Authors:** Kumari Shubha, Arbind Kumar Choudhary, Abhishek Kumar Dubey, Kuldeep Tripathi, Rakesh Kumar, Santosh Kumar, Anirban Mukherjee, Manisha Tamta, Ujjwal Kumar, Sanjeev Kumar, Jayanta Layek, Anup Das

**Affiliations:** ^1^ICAR Research Complex for Eastern Region, Patna, Bihar, India; ^2^ICAR National Bureau of Plant Genetic Resources, New Delhi, India; ^3^ICAR Research Complex for NEH Region, Umiam, Meghalaya, India

**Keywords:** lablab bean, nutritional components, path coefficient analysis, correlation, collar rot

## Abstract

**Introduction:**

Malnutrition continues to be a significant concern at unacceptably high levels globally. There is significant potential for addressing malnutrition of human population through the biofortification of climate-resilient vegetables using strategic breeding strategies. Lablab bean [*Lablab purpureus* (L.) Sweet], a underutilized nutrient-dense crop holds great potential in this aspect. Despite its advantageous nutritional profile, the production, research, and consumption of lablab bean are currently limited. Addressing these limitations and unlock the nutritional benefits of lablab beans needs to prioritized for fighting malnutrition in local inhabitants on a global scale.

**Materials and methods:**

Twenty five genotypes of lablab bean collected through exploration survey in Eastern India and were evaluated in 2020–2021. Among them, the nine highly diverse well adapted genotypes were again evaluated at the experimental farm of ICAR-Research Complex for Eastern Region, Patna, Bihar, India in 2021–2022. Horticultural important traits of lablab bean were recorded by using the minimum descriptors developed by ICAR-NBPGR in New Delhi and biochemical analysis was done by using standard protocols. Genotypic and phenotypic correlation and path coefficient analysis was done used understand relationships, interdependencies, and causal pathways between different traits. The outcome was revalidated by using principal component analysis (PCA).

**Results:**

Descriptive statistics revealed substantial heterogeneity across the traits of lablab bean evaluated. Vitamin A content showed nearly a five-fold variation, Fe ranged from 5.97 to 10.5 mg/100 g, and Vitamin C varied from 4.61 to 9.45 mg/100 g. Earliness and dwarf growth was observed in RCPD-1 (60 cm) and early flowering (41 days). RCPD-3 and RCPD-12 had high pod yield due to their high number of pods and pod weight. Pod yield was significantly correlated with number of pod per plant (NPP) (*r_g_* = 0.995) and with average pod weight (APW) (*r_g_* = 0.882). A significant positive correlation was also found between protein and Zn content (*r_g_* = 0.769). Path coefficient analysis revealed that average pod weight had the most direct positive effect on pod yield, followed by NPP and protein content. The reaction of lablab bean genotypes to collar rot disease was also evaluated and significant differences in disease intensity were observed among the genotypes, with the resistant check RCPD-15 exhibiting the lowest disease intensity.

**Discussion:**

The study highlights the substantial heterogeneity in lablab bean traits, particularly in nutritional components such as vitamin A, iron, and vitamin C concentrations. Early flowering and dwarf growth habit are desirable qualities for lablab bean, and certain genotypes were found to exhibit these traits. Positive correlations, both phenotypic and genotypic, existed among different traits, suggesting the potential for simultaneous improvement. Path coefficient and PCA revealed genotypes with high yield and nutritional traits. Finally, resistant and moderately resistant lablab bean genotypes to collar rot disease were identified. These findings contribute to the selection and breeding strategies for improving lablab bean production and nutritional value.

## 1 Introduction

The Sustainable Development Goal Target 2.2 calls for the abolition of all forms of malnutrition by 2030 (1, 2). However, simply increasing access to staple food grains will not solve this problem. It demands using lesser-known nutrient rich plant species in the diet, which have low input requirements and are locally available (3). Advancements in research and enhancement of nutri-dense underutilized species are crucial for ensuring nutritional security within indigenous communities in their respective habitats. Lablab bean [*Lablab purpureus* L. (Sweet)], also referred to as Hyacinth bean, Dolichos bean, or Indian bean, is a leguminous vegetable crop that has become increasingly popular in recent times due it’s multifunctional benefits like generating income for resource-poor individuals, nutritional superiority, soil conservation properties and climate-smart crop (4). It is a rich source of vegetable proteins, with its seeds and pods containing 20–28% protein, making it an excellent source of nutrition (5, 6). In addition to its protein content, it is also rich in carbohydrates (60.74 g), fats (1.69 g), fibers (25.6 g), and minerals such as iron (Fe) (5.1 mg), phosphorus (P) (0.372 mg) and zinc (9.3 mg) making it a well-rounded and nutritious food (7). Furthermore, the lablab bean contains nutraceutical compounds such as Chikusetsusaponin IV A and glucosides, as well as protein isolate, which can be used as flavor enhancers to improve cake quality (8–11). Its versatility extends beyond its use as a food source as it can also be used for forage, nitrogen-fixation, and even as a biocontrol agent for pests (7). Recent research has shown that lablab bean extracts have the ability to hinder viral infections, including influenza and SARS-CoV-2, which was responsible for global pandemic (12). These benefits highlight the importance of enhancing the production and exploitation of lablab bean.

In India, the total area under beans is 0.228 mha with an annual production of 2.51 mT while in eastern India the annual production of bean is 0.93 Mt (NHB-2021-22). Despite the facts that lablab bean is a promising climate-smart crop, particularly beneficial for protein-dependent rural communities in Asia and Africa (13), its productivity has not increased significantly. It is consequently important to increase crop yield in order to meet rising needs from an expanding human population.

Lablab bean breeding programs aim to develop both new varieties and potential parents with desirable traits which can lead to increased productivity and crop production (13). However, most of the desirable traits like pod yield and nutritional traits are complex and inclined by a range of reasons, including polygenes and environmental conditions (14). Focusing solely on complex traits like yield and particular one nutritional trait is not sufficient for selecting higher yields and nutritional traits due to its complexity and linkage with other associated traits. Therefore, it is important to consider other interrelated traits in lablab bean (15). The relationship between lablab bean traits can be studied using correlation analysis, and more precisely through path coefficient analysis, which is a statistical approach that allows for the partitioning of correlation coefficients into direct and indirect effects (16). These studies enable the evaluation of the interconnections among different traits and aiding in the selection of multiple traits. Correlation and path coefficient analysis is commonly used by researchers in many crop including lablab bean (17), cowpea (14), Bambara groundnut (18), common bean (19), and garden pea (20). However, no study has investigated exclusively the combined phenotypic and genetic correlations and path coefficient analysis among pod yield and nutritional components in lablab bean. Accurately comprehending the relationship between the nutritional quality traits and pod yield components of lablab bean could lead to more efficient identification and selection of superior genotypes that exhibit both high pod yield and enhanced nutritional traits. The successful execution of the “HarvestPlus” program by CGIAR has confirmed the effectiveness of conventional breeding in developing biofortified crops like sweet potato, rice, cassava, and millets. These crops have proven beneficial in addressing mineral deficiencies and improving the health of targeted populations by enriching them with essential nutrients such as vitamin A, iron, and zinc (Zn) (21). In a similar vein, assessing genetic variants in genotypes or breeding lines offers intriguing opportunities for generating biofortified lablab beans with increased protein content, iron (Fe), Zn, and other essential minerals. This approach has the potential to improve the nutritional value of lablab beans as well as their contribution to human health (22).

In order to address the low production of lablab beans, one of the significant biotic factors is the collar rot disease caused by *Sclerotium rolfsii* in India (23). This disease is especially problematic when prevailing weather condition is favorable for pathogen growth, such as good soil moisture, high soil temperatures ranging between 25 and 30°C, and low organic matter in the soil (24). Collar rot is a devastating disease due to its wide host range, rapid growth of its pathogen, and the production of persistent sclerotia, which leads to significant economic losses (23). To address this issue, a preliminary evaluation of various genotypes was conducted to identify potential resistance source to collar rot.

Based on our literature review, there is a notable lack of reliable and sufficient data relative to the nutrient composition of lablab bean genotypes, as well as its link with pod yield-related parameters and resistance to collar rot disease. Taking into account the aforementioned background information as well as the paucity of data on the nutrient content of lablab bean genotypes grown in eastern India, the current research was undertaken.

## 2 Materials and methods

### 2.1 Experimental material and data collection

**Figure 1:**
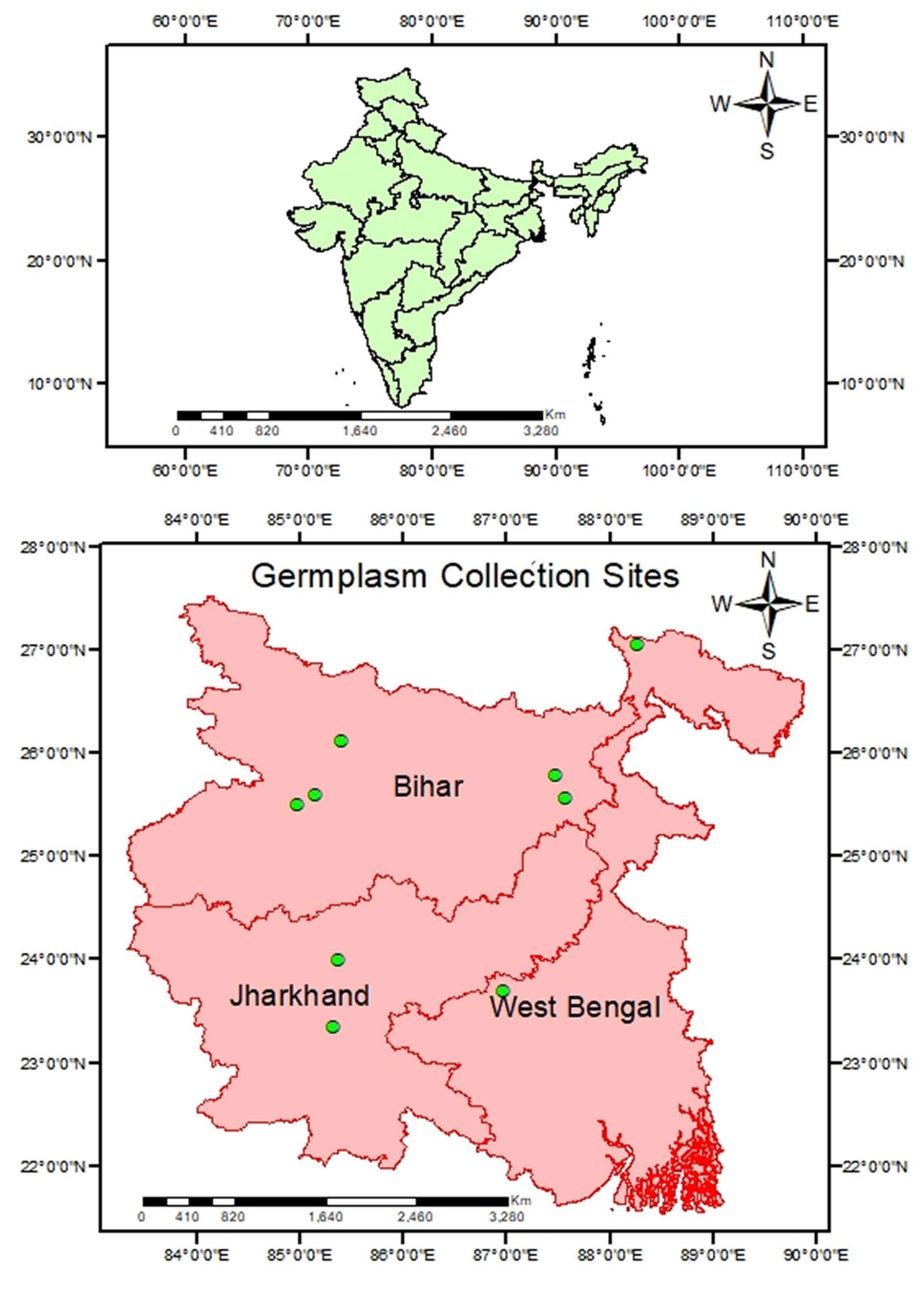
Map showing germplasm collection site of lablab bean.

Twenty-five genotypes, collected through exploration survey in Eastern India, were evaluated at the experimental farm of ICAR-Research Complex for Eastern Region, Patna, Bihar, India by following augmented block design in 2020–2022, among which nine highly diverse in growth habits, pod size, pigmentation and well adapted to Eastern Indian condition were selected to study (Figure 1). In this experimental design, due to limited experiment materials, it’s not possible to replicate the experiment materials for the test treatment. However, we have optimized the efficiency of each observation by maximizing the replication of control treatments within each block for effective comparison with the test treatment. Photo and thermo sensitivity of selected genotypes was determined through a seasonal growth experiment (mentioned in Supplementary Table S1). These genotypes were cultivated during both winter and summer seasons, which have varying day lengths and temperatures. While photo-insensitive genotypes flowered in both seasons, during the summer, the high temperatures hindered successful fruit set, leading to their characterization as thermo-sensitive. Recommended agronomical practices were followed, including plot preparation, fertilization, and application of fungicides and insecticides (mentioned in Supplementary Table S2). To ensure individuality of each genotype, care was taken to segregate them properly. To prevent the influence of border effects, morphological features were measured on 10 randomly chosen plants from each genotype, and the average was reported. Tagging allowed the selected plants to be recognized at an early stage of development. Diversifying qualitative traits like plant growth habit, pigmentation on stem, leaf color, flower color, pigmentation on pod, photosensitivity, thermo-sensitivity and selected quantitative traits including days to 50% flowering (D50%F), plant height (in cm) (PH), number of pods per plant (NPP), number of seeds per pod (NSP), average pod weight (APW) (g), pod length (PL) (cm), days to 1st picking (DFP), and pod yield per plant (g) were measured using the minimum descriptors developed by ICAR-NBPGR in New Delhi. Agronomic traits of 25 genotypes are presented in Supplementary Table S3. For analysis, the immature pods (which serves as vegetable purpose) of each genotype were harvested. To minimize moisture content, the seeds were manually shifted and washed before being oven-dried at 60°C for 48 h. The dried pods were pulverized in a lab seed grinder and sieved through a 0.5 mm sieve. A 0.5 g flour sample was collected from the milled seeds for examination.

### 2.2 Estimation of nutritional components

Protein content was determined using the Automated Biokjgel Protein Estimation Machine (IS: 7219:1973 RA 2005). A test portion of 0.5–1.00 g was weighed and placed in a digestion tube along with 30 g potassium sulfate, 0.5 g anhydrous cupric sulfate, and 10 mL concentrated H_2_SO_4_. The digestion followed a temperature program: 250°C for 10 min, 300°C for 10 min, 350°C for 10 min, and 420°C for 75 min. After cooling, automatic distillation was carried out with 40% NaOH and 4% Boric acid. Manual titration with 0.1 N HCl/0.1 N H_2_SO_4_ was performed, with the endpoint indicated by a pink color, confirming protein levels through Kjeldahl’s method. The process involved sample preparation, digestion, distillation, and titration steps, following specified procedures and chemicals.

Vitamin A and Vitamin C analysis were conducted using HPLC, following the QA.16.5.3 and IS: 5838–1970 (RA-2005) methods, respectively. The minerals, including Iron (Fe), Zinc (Zn), and Calcium (Ca), were estimated using the Inductively Coupled Plasma Mass Spectrometry (ICP-MS) (Model: PerkinElmer’s NexION^®^2000, United States) (QA.16.5.2) method. Phosphorus (P) was estimated using UV–Visible spectrophotometer (Model: UV 1800, Shimadzu, Japan), following the method described in IS: 14828:2000.

### 2.3 Screening of lablab bean genotypes against collar rot under field conditions

To screen for collar rot disease, isolates were collected from plants exhibiting typical symptoms of mycelia growth on the collar zone adjacent to the soil level. The soil was infected with an infestation of sclerotia, which are compact masses of fungal mycelium and hyphae that can overwinter and serve as a source of infection. The pathogenic microorganism *S. rolfsii* exhibits cottony white colony morphology when cultured on PDA (Potato Dextrose Agar). These colonies display a mycelial growth ranging from dull white to pure white, and the development of sclerotia typically commences after approximately 8–9 days of incubation. The sclerotia, which are brown in color and resemble mustard seeds in shape, serve as distinctive identifying features for this pathogen. Based on these distinctive characteristics, the pathogen was conclusively identified as *S. rolfsii*.

For disease reaction, the initial plant stand upon germination was measured 20 days after seeding, and the final survival was measured throughout flowering and harvesting. The % disease intensity of wilted plants was calculated using the difference between the starting and final plant populations of various genotypes. The percentage infection incidence was determined using Wheeler (25) method.


$$\mathrm{Percent}\;\mathrm{disease}\;\mathrm{incidence}\;\left(PDI\right)=\frac{\mathrm{Number}\;\mathrm{of}\;\mathrm{diseased}\;\mathrm{plants}}{\mathrm{Total}\;\mathrm{number}\;\mathrm{of}\;\mathrm{plants}\;\mathrm{observed}}\times 100$$

The genotypes were categorized into various levels of susceptibility and resistance based on their mortality percentage, using the standard rating scale developed by ICRISAT (26) (mentioned in Supplementary Table S4).

### 2.4 Statistical analysis

The mean, range, standard deviation (SD), coefficient of variation (CV), standard error, and skewness were calculated using the IBM Statistical Package for the Social Sciences (SPSS) software (version 20.0). Genotypic and phenotypic correlation coefficients, as well as path coefficient analysis, were calculated using SAS 9.4 (Statistical Analysis System) software (SAS Institute Inc., Cary, NC, United States). The Pearson’s correlation heatmap and PCA component loading plots were constructed using a web interface (MetaboAnalyst) (27).

## 3 Results

The descriptive statistical parameters for different characters are presented in Table 1. The coefficient of variation (CV) for yield and nutritional components varied from 8.68% (protein) to 47.08% (vitamin A), showing that there is substantial heterogeneity across the traits evaluated. The protein concentration in the genotypes was 19.01–25.5 g/100 g, indicating that variation within a genotype was only 1.34-fold. Among micronutrients, vitamin A ranged from 6.26 to 30.85 mcg/100 g, indicating nearly fivefold variation in the tested genotypes. Similarly, Fe ranged 5.97–10.5 mg/100 g and vitamin C from 4.61 to 9.45 mg/100 g, indicating twofold variation for the Fe and vitamin C, both. Earliness is a highly desirable quality in vegetables in the sense that the prevailing prices in the market are invariably very high in early season. The genotype RCPD-1 was identified as earliest for time taken to 50% flowering (41 days) followed by RCPD-2 and RCPD-16 for taking 45 days each to reach 50% flowering. Moreover, various genotypes were assessed for distinct plant characteristics, specifically plant height and pod yield. Genotype RCPD-1 exhibited the lowest plant height at 60 cm, followed by RCPD-6 and RCPD-16 at 65 cm each. Notably, RCPD-3 and RCPD-12 demonstrated a high pod yield attributed to a combination of an indeterminate growth habit, resulting in prolonged pod production, and a substantial number of pods with high individual pod weight. Furthermore, within the bush type genotypes, RCPD-1 and RCPD-16 were identified as prolific bearers and high yielders. These genotypes epitomize an ideotype characterized by a semi-determinate growth pattern, early flowering habits, and superior pod quality suitable for vegetable purposes. Notably, this ideotype obviates the need for cost-intensive trellising methods.

**Table 1:** Estimates of variance for the traits studied in lablab bean genotypes.

	Days to 50% flowering	Plant height (cm)	No. of pod per plant	No. of seed per pod	Avg pod weight (g)	Pod length (cm)	Days to 1st picking	Pod yield per plant (g)	Protein (g)	Vit C (mg)	Vit A (mcg)	Zinc (mg)	Iron (mg)	Calcium (mg)	Phosphorous (mg)
Min	40.67	60	58	4.33	5.33	8	57	373	19.01	4.61	6.27	1.70	5.97	321.6	317.89
Max	57.33	154	145	6.67	9.23	13.67	75.67	997	25.47	9.45	30.86	2.59	10.50	459.1	460.85
Mean	50.93	110.11	91.56	5.48	6.19	11.19	68.19	593.22	21.80	6.12	17.23	2.28	7.92	395.3	363.89
Skewness	−0.72	−0.28	0.67	0.18	2.44	−0.50	−0.71	−0.28	0.74	1.62	0.26	−0.84	0.93	−0.27	1.24
Kurtosis	−0.89	−2.08	−0.91	−0.38	6.30	−0.93	−0.89	−1.56	0.93	3.77	−0.71	−0.29	−0.01	−1.13	1.19
Std. error	1.98	12.84	10.66	0.24	0.40	0.64	2.16	73.94	0.63	0.48	2.70	0.10	0.50	16.86	15.55
Stand. Dev.	5.94	38.53	31.97	0.73	1.21	1.92	6.49	221.81	1.89	1.44	8.11	0.31	1.49	50.58	46.66
Coeff. Var.	11.66	34.99	34.92	13.29	19.55	17.16	9.51	37.39	8.68	23.55	47.08	13.53	18.84	12.79	12.82

### 3.1 Phenotypic correlations for pod yield components and nutritional quality traits

**Figure 2:**
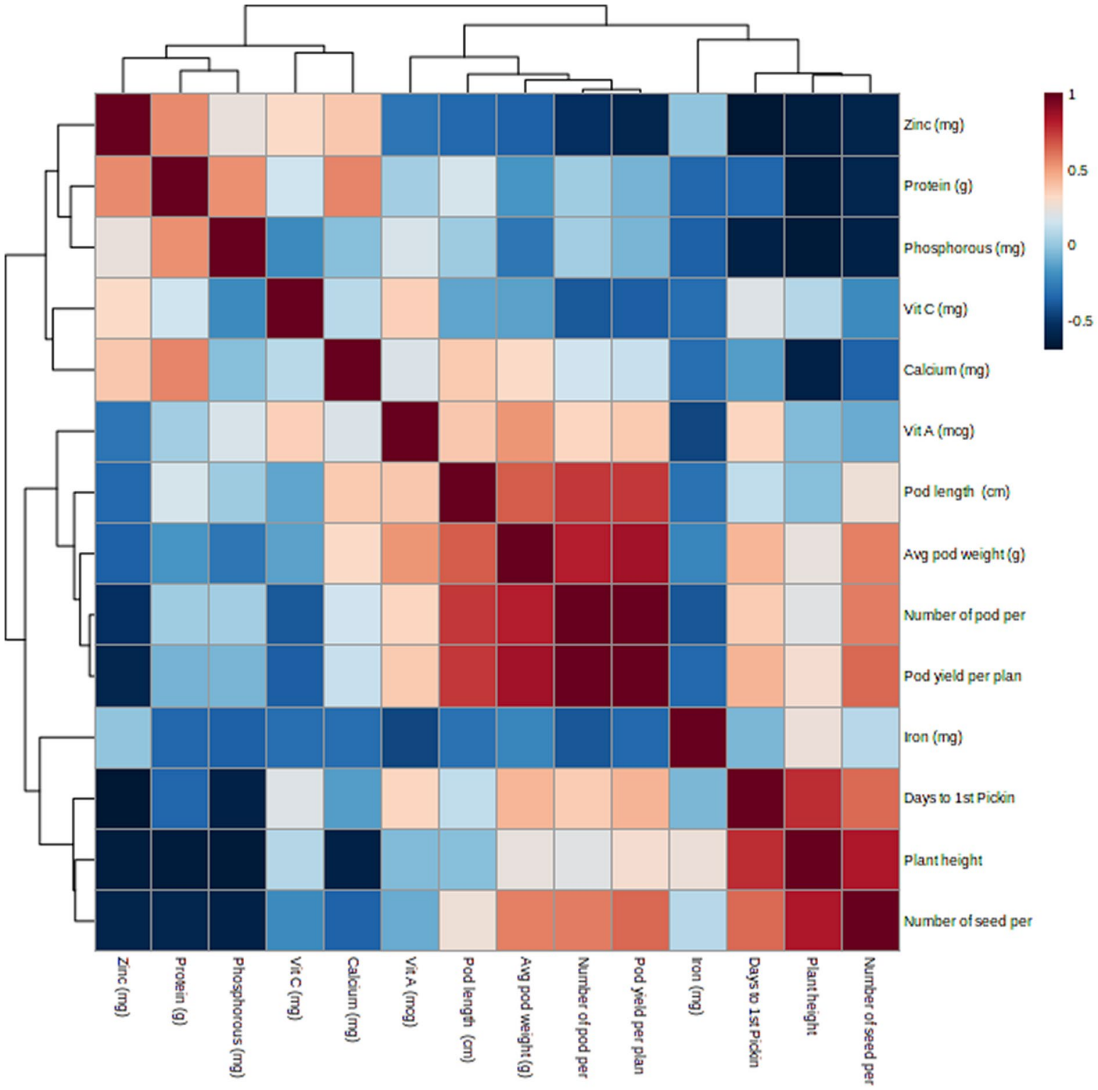
Heat map representing phenotypic correlation between lablab bean pod yield components and nutritional quality traits.

Phenotypic correlation is a way for determining the relationship between two sets of phenotypic values or variables in a population (as shown in Table 2), but it does not indicate the strength of the relationship. A heatmap was used to visualize the correlation between traits (as shown in Figure 2). Highly significant (*p* < 0.001) positive correlations were found for D50F with DFP (*r_p_* = 0.964) and NPP with pod yield (*r_p_* = 0.991). Significant (*p* < 0.01) positive correlations were found for D50F with PH (*r_p_* = 0.806), APW with NPP (*r_p_* = 0.825), protein with Zn (*r_p_* = 0.855), and APW (*r_p_* = 0.863) with pod yield. Significant (*p* < 0.05) positive correlations were found for PL with NPP (*r_p_* = 0.755), APW with Vit A (*r_p_* = 0.729), protein with Ca (*r_p_* = 0.671), protein with P (*r_p_* = 0.28) and Vit C with Zn (*r_p_* = 0.669). Significant (*p* < 0.01) negative correlations were found for PH with NSP (*r_p_* = −0.839) and significant (*p* < 0.05) negative correlations were found for DFP with Zn (*r_p_* = −0.680). The heat map showed strong positive correlations between pod yield and number of pods per plant (NPP), average pod weight and pod length (Figure 2).

**Table 2:** Phenotypic correlations among lablab yield components and nutritional quality traits.

	Days to 50% flowering	Plant height	Number of pod per plant	Number of seed per pod	Average pod weight	Pod length	Days to 1st picking	Protein	Vit C	Vit A	Zn	Fe	Ca	P	Pod yield per plant
Days to 50% flowering	1														
Plant height	0.806**	1													
No. of pod per plant	0.265	0.211	1												
No. of seed per pod	0.571	−0.839**	0.586	1											
Avg pod weight (g)	0.358	0.242	0.825**	0.582	1										
Pod length (cm)	0.056	−0.027	0.755*	0.271	0.668*	1									
Days to 1st picking	0.964***	0.78	0.375	0.637	0.437	0.119	1								
Protein (g)	−0.449	−0.644	0.039	−0.578	−0.163	0.176	−0.341	1							
Vit C (mg)	0.218	0.083	−0.373	−0.204	−0.124	−0.112	0.203	0.154	1						
Vit A (mcg)	0.414	−0.04	0.347	−0.092	0.729*	0.387	0.338	0.041	0.359	1					
Zn	−0.736*	−0.634	−0.506	−0.587	−0.358	−0.327	−0.680*	0.855**	0.669*	−0.28	1				
Fe	0.056	0.263	−0.389	0.093	−0.224	−0.295	−0.051	−0.335	−0.308	−0.441	−0.01	1			
Ca	−0.298	−0.611	0.176	−0.349	0.718*	0.381	−0.139	0.671*	0.1	0.194	0.387	−0.308	1		
P	−0.543	−0.648	0.032	−0.603	−0.278	0.025	−0.604	0.745*	−0.202	0.182	0.248	−0.358	−0.026	1	
Pod yield per plant	0.352	0.292	0.991***	0.644	0.863**	0.753*	0.444	−0.068	−0.361	0.380	−0.577	−0.328	0.135	−0.057	1

***p < 0.001, **p < 0.01, *p < 0.05.

### 3.2 Genetic correlations for pod yield components and nutritional quality trait

Genetic correlation is a method used to measure the genetic relationship between two traits by assessing additive genetic variation, aiding botanists in understanding trait inheritance. This study revealed that there was a strong and positive correlation (*r_g_* = 0.995) between pod yield and NPP, which was highly significant (*p* < 0.001) and documented (Table 3). Significant (*p* < 0.01) positive correlations were found for NPP (*r_g_* = 0.838) with APW and APW (*r_g_* = 0.882) with pod yield. Significant (*p* < 0.05) positive correlations were found for NPP with PL (*r_g_* = 0.769), PL with APW (*r_g_* = 0.690), protein with Zn (*r_g_* = 0.769) and pod yield with NSP (*r_g_* = 0.730) and PL (*r_g_* = 0.767). However, highly significant (*p* < 0.001) negative correlations were found for PH with NSP (*r_g_* = −0.937). The correlation results revealed that the genotypic level is more closely related to the associated phenotypic level in the majority of cases.

**Table 3:** Genetic correlations among lablab bean genotype yield components and nutritional quality traits.

	Days to 50% flowering	Plant height (cm)	Number of pod per plant	Number of seed per pod	Average pod weight	Pod length	Days to 1st Picking	Protein	Vit C	Vit A	Zn	Fe	Ca	P	Pod yield per plant
Days to 50% flowering	1														
Plant height	0.823**	1													
Number of pod per plant	0.096	0.214	1												
Number of seed per pod	0.538	−0.937**	0.671*	1											
Average pod weight	0.1	0.247	0.838**	0.637	1										
Pod length	0.084	−0.031	0.769*	0.325	0.690*	1									
Days to 1st picking	0.981**	0.804**	0.173	0.592	0.099	−0.062	1								
Protein	−0.413	−0.645	0.035	−0.646	−0.166	0.180	−0.281	1							
Vit C	0.237	0.083	−0.380	−0.228	−0.127	−0.114	0.227	0.154	1						
Vit A	0.258	−0.039	0.342	−0.103	0.738*	0.394	0.124	0.0411	0.360	1					
Zn	−0.714*	−0.634	−0.514	−0.655	−0.365	−0.334	−0.687*	0.756*	0.319	−0.280	1				
Fe	0.123	0.263	−0.387	−0.103	−0.228	−0.300	0.026	−0.334	−0.308	−0.440	−0.01	1			
Ca	−0.437	−0.611	0.166	−0.389	0.323	0.389	−0.305	0.571	0.1	0.194	0.388	−0.308	1		
P	−0.489	−0.649	0.035	0.673*	−0.282	0.025 ns	−0.542	0.544	−0.202	0.182	0.248	−0.358	−0.026	1	
Pod yield per plant	0.171	−0.293	0.995**	0.730*	0.882**	0.767*	0.226	−0.068	−0.351	0.381	−0.576	−0.328	0.135	−0.057	1

**p < 0.01, *p < 0.05.

### 3.3 Path coefficient analysis for pod yield and yield components

In the process of selective breeding for greater pod yield, it is important to take into account multiple characteristics that enhance yield instead of solely concentrating on yield, which is influenced by other traits, environment and is a complex feature. Path coefficient analysis is a dependable statistical technique that can assist in the selection process by breaking down correlation coefficients into direct and indirect effects. This helps breeders to identify the most influential characteristics to consider in their breeding program.

#### 3.3.1 Direct and indirect effects of yield components and nutritional components on pod yield

**Figure 3:**
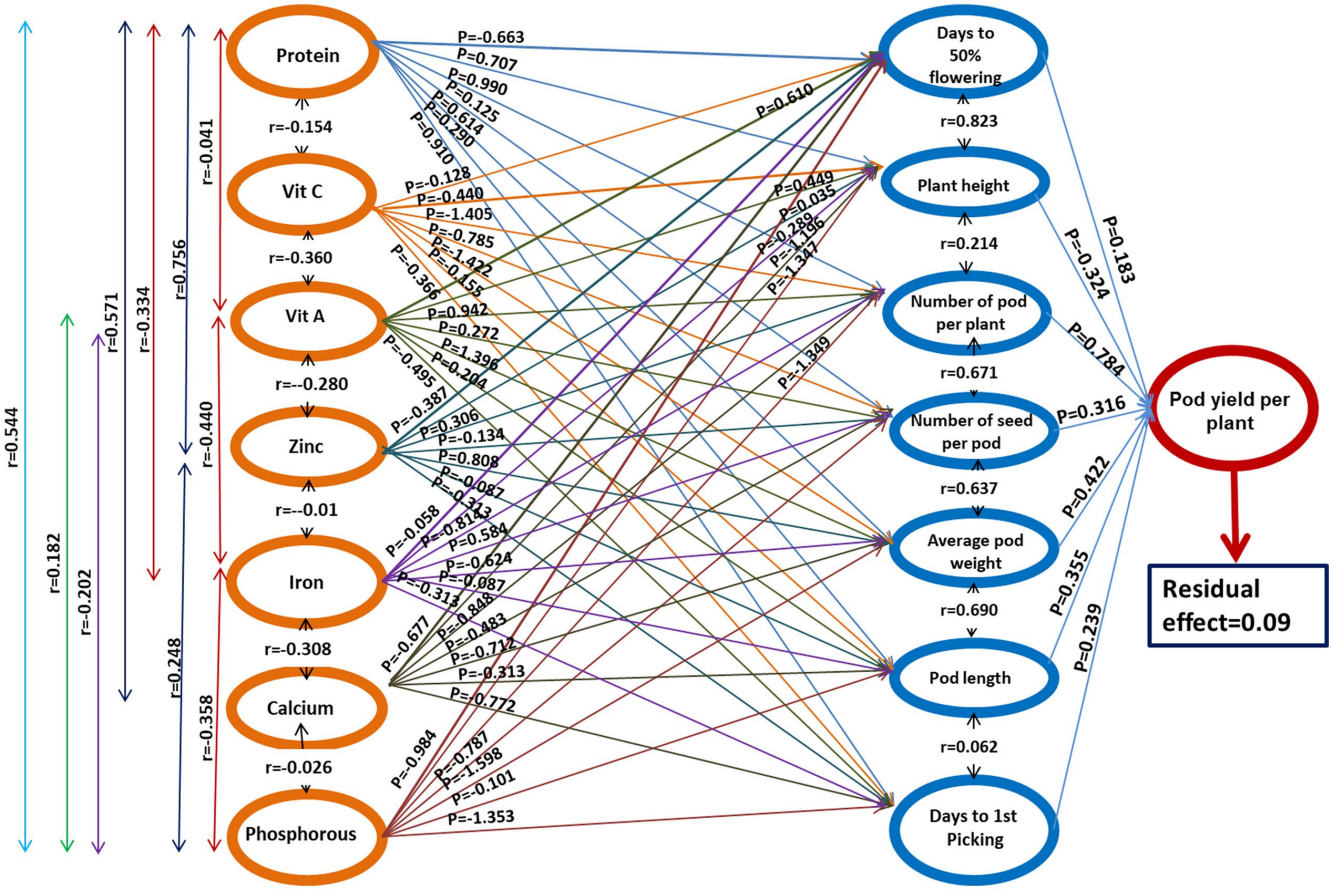
The genotypic path coefficient diagram illustrates the causal relationships among quantitative traits and pod yield. The direct effects are represented by single arrowed lines and mutual connections are depicted by double arrowed lines. The coefficients of factors and their correlation coefficients are represented as P and r, respectively.

The path coefficient is a standardized regression coefficient that measures the strength and direction of relationships in a model, determining direct and indirect effects. In the study, path coefficient analysis was employed to examine the combined impact of yield components and nutritional quality traits on pod yield. As shown in Figure 3 and Table 4, pod yield is an outcome of all contributing factors such as D50%F, PH, NPP, NSP, APW, PL, DFP, protein, Vitamin C, Vitamin A, Zn, Fe, Ca, and P. The correlation coefficients of these contributing traits with pod yield were analyzed as direct and indirect effects (as illustrated in Figure 3). In our study, the average pod weight had the most direct positive effect on the pod yield per plant (0.724), followed by NPP (0.480) and protein (0.258). The NPP had the highest indirect influence on pod yield followed by APW (0.882), NPP (0.767), and PL (0.730).

**Table 4:** The direct (diagonal) and indirect effects of 14 characteristics on pod yield in lablab bean genotypes.

Traits	Days to 50% flowering	Plant height	Number of pod per plant	Number of seed per pod	Avg pod weight	Pod length	Days to 1st Picking	Protein	Vit C	Vit A	Zinc	Iron	Calcium	Phosphorous
Days to 50% flowering	**−0.542**	−0.437	−0.144	−0.310	−0.194	−0.030	−0.523	0.243	−0.118	−0.225	0.399	−0.030	0.162	0.294
Plant height	0.264	**−0.327**	0.069	0.275	0.079	−0.009	0.255	−0.211	0.027	−0.013	−0.207	0.086	−0.200	−0.212
Number of pod per plant	0.127	0.101	**0.480**	0.281	0.396	0.362	0.180	0.019	−0.179	0.167	−0.243	−0.187	0.084	0.015
Number of seed per pod	−0.140	−0.206	−0.144	**−0.246**	−0.143	−0.067	−0.157	0.142	0.050	0.023	0.144	−0.023	0.086	0.148
Avg pod weight (g)	0.259	0.175	0.597	0.421	**0.724**	0.483	0.316	−0.118	−0.090	0.383	−0.259	−0.162	0.230	−0.201
Pod length (cm)	−0.008	0.004	−0.114	−0.041	−0.101	**−0.151**	−0.018	−0.027	0.017	−0.058	0.049	0.044	−0.058	−0.004
Days to 1st Picking	0.090	0.073	0.035	0.060	0.041	0.011	**0.094**	−0.032	0.019	0.032	−0.064	−0.005	−0.013	−0.057
Protein (g)	−0.116	−0.166	0.010	−0.149	−0.042	0.045	−0.088	**0.258**	0.040	0.011	0.143	−0.086	0.147	0.140
Vit C (mg)	0.026	0.010	−0.045	−0.024	−0.015	−0.013	0.024	0.018	**0.120**	0.043	0.038	−0.037	0.012	−0.024
Vit A (mcg)	−0.060	0.006	−0.050	0.013	−0.076	−0.056	−0.049	−0.006	−0.052	**−0.144**	0.040	0.063	−0.028	−0.026
Zinc (mg)	0.440	0.379	0.302	0.351	0.214	0.196	0.406	−0.332	−0.191	0.167	**−0.598**	0.006	−0.231	−0.148
Iron (mg)	−0.001	−0.003	0.005	−0.001	0.003	0.004	0.001	0.004	0.004	0.006	0.000	**−0.013**	0.004	0.005
Calcium (mg)	0.018	0.036	−0.010	0.021	−0.019	−0.023	0.008	−0.034	−0.006	−0.012	−0.023	0.018	**−0.060**	0.002
Phosphorous (mg)	−0.006	−0.007	0.000	−0.006	−0.003	0.000	−0.006	0.006	−0.002	0.002	0.003	−0.004	0.000	**0.011**
Pod yield per plant (g)	0.171	−0.293	0.995**	0.730*	0.882**	0.767*	0.226	−0.068	−0.351	0.381	−0.576	−0.328	0.135	−0.057

Residual effect: 0.09.

Bold and diagonal lines represent direct effects. **p < 0.01, *p < 0.05.

#### 3.3.2 Relationships in nutritional components and yield components

The nutritional components, namely protein, vitamin C, vitamin A, Zn, Fe, Ca and were classified as first-order components. Meanwhile, the second-order components included yield components such as D50F, PH, NPP, NSP, PL, APW, and DFP. These yield components were considered critical factors in determining the overall yield of lablab beans.

#### 3.3.3 The effects of a first-order component association on a second-order component

The effects of the first-order component (nutritional parameters) on the days to 50% flowering (Table 5) revealed that only vitamin A had a strong positive association (0.610), whereas strong negative correlation with protein (−0.663) and Ca (−0.677). A similar trend was observed for PH, results revealed strong positive association (0.610) with Vitamin A, whereas strong negative correlation with protein (−0.663). The NPP had strong positive association with protein (0.990) and vitamin A (0.942), whereas strong negative correlation with Fe (−0.814) and Ca (−0.848).

**Table 5:** First order and second-order component relationships.

Components	Traits	Protein	Vit C	Vit A	Zn	Fe	Ca	P
Days to 50% flowering	Protein	**−0.663**	−0.019	0.025	−0.214	0.019	−0.386	−0.535
	Vit C	0.102	**−0.128**	0.219	−0.123	0.017	−0.067	0.198
	Vit A	0.027	−0.046	**0.610**	0.108	0.026	−0.131	−0.179
	Zn	0.368	−0.041	−0.171	**−0.387**	0.001	−0.262	−0.244
	Fe	−0.222	0.040	−0.269	0.004	**−0.058**	0.209	0.352
	Ca	0.379	−0.013	0.118	−0.150	0.018	**−0.677**	0.026
	P	0.361	0.026	0.111	−0.096	0.021	0.018	**−0.984**
		**−0.413**	**0.237**	**0.258**	−0.714*	**0.123**	**−0.437**	**−0.489**
Plant height	Protein	**−0.707**	−0.068	0.018	0.019	0.097	−0.683	−0.734
	Vit C	0.109	**−0.440**	0.162	0.011	0.089	−0.120	0.272
	Vit A	0.029	−0.158	**0.449**	−0.010	0.127	−0.232	−0.245
	Zn	0.392	−0.140	−0.126	**0.035**	0.003	−0.463	−0.334
	Fe	−0.236	0.135	−0.198	0.000	**−0.289**	0.369	0.483
	Ca	0.404	−0.044	0.087	0.013	0.089	−**0.196**	0.035
	P	0.385	0.089	0.082	0.009	0.104	0.031	**−0.347**
		**−0.645**	**−0.083**	**−0.039**	**−0.634**	**0.263**	**−0.611**	**−0.649**
NPP	Protein	**0.990**	−0.216	0.039	0.170	0.272	−0.484	−0.735
	Vit C	0.153	**−0.405**	0.339	0.097	0.250	−0.085	0.272
	Vit A	0.041	−0.505	**0.942**	−0.086	0.359	−0.165	−0.246
	Zn	0.549	−0.448	−0.264	**0.306**	0.008	−0.328	−0.335
	Fe	−0.331	0.432	−0.415	−0.003	**−0.814**	0.261	0.483
	Ca	0.566	−0.141	0.183	0.118	0.251	**−0.848**	0.035
	P	0.539	0.284	0.172	0.076	0.292	0.022	**0.349**
		0.035	−0.380	0.342	−0.514	−0.387	0.166	0.035
NSP	Protein	**0.125**	−0.107	−0.010	0.639	0.139	−0.950	−1.413
	Vit C	0.067	**−0.785**	0.401	0.376	0.067	0.265	0.427
	Vit A	−0.009	−0.563	**0.272**	−0.332	0.240	−0.196	−0.490
	Zn	0.635	−0.592	−0.372	**−0.134**	−0.009	−0.779	−0.603
	Fe	−0.268	0.206	−0.523	0.017	**0.584**	0.006	1.194
	Ca	0.599	0.265	0.140	0.495	0.002	**−0.483**	−0.077
	P	0.666	0.320	0.261	0.286	0.292	−0.057	**0.787**
		−0.646	−0.228	0.103	−0.655	−0.103	−0.389	0.673*
APW	Protein	**0.619**	−0.219	0.057	0.448	0.209	−0.407	−0.870
	Vit C	0.095	**−1.422**	0.501	0.259	0.192	−0.071	0.323
	Vit A	0.025	−0.511	**0.996**	−0.226	0.275	−0.138	−0.291
	Zn	0.343	−0.455	−0.390	**0.808**	0.006	−0.275	−0.395
	Fe	−0.207	0.438	−0.615	−0.008	**−0.624**	0.220	0.573
	Ca	0.354	−0.142	0.271	0.313	0.193	**−0.712**	0.042
	P	0.337	0.287	0.254	0.200	0.224	0.019	**−0.598**
		−0.166	−0.127	0.738*	−0.365	−0.228	0.323	−0.282
PL	Protein	**0.290**	−0.024	0.008	−0.275	0.029	0.202	−0.055
	Vit C	0.045	**−0.155**	0.073	−0.158	0.027	0.035	0.020
	Vit A	0.012	−0.056	**0.204**	0.138	0.038	0.069	−0.018
	Zn	0.161	−0.049	−0.057	**−0.495**	0.001	0.137	−0.025
	Fe	−0.097	0.048	−0.090	0.005	**−0.087**	−0.109	0.036
	Ca	0.166	−0.016	0.040	−0.192	0.027	**0.354**	0.003
	P	0.158	0.031	0.037	−0.123	0.031	−0.009	**−0.101**
		0.180	−0.114	0.394	−0.334	−0.3	0.389	0.025
DFP	Protein	**−0.910**	−0.056	0.025	−0.147	0.105	−0.441	−0.737
	Vit C	0.140	**−0.366**	0.221	−0.084	0.096	−0.077	0.273
	Vit A	0.037	−0.132	**0.616**	0.074	0.138	−0.150	−0.247
	Zn	0.505	−0.117	−0.172	**−0.265**	0.003	−0.299	−0.336
	Fe	−0.305	0.113	−0.272	0.003	**−0.313**	0.238	0.485
	Ca	0.520	−0.037	0.120	−0.102	0.096	**−0.772**	0.036
	P	0.496	0.074	0.112	−0.066	0.112	0.020	**−0.353**
		−0.281	0.227	0.124	−0.687*	0.026	−0.305	−0.542

Bold and diagonal lines represent direct effects. *p < 0.05.

The effects of the first-order component on the total number of seeds per pod revealed that Fe (0.584) and *p* (0.787) had positive correlation while vitamin C (−0.785) and Ca (−0.483) had a negative association NSP, whereas all other components had a positive correlation. The APW (0.519) had the strongest positive correlation with the vitamin A (0.996) followed by Zn (0.808) and protein (619), while negative correlation with Ca (−0.712), Fe (−0.624), P (−0.598). When conducting path analysis of the first-order component with pod length, it was found that Zn had a negative direct impact (−0.495), while Ca had a positive direct impact (0.354). Additionally, the analysis showed that the first-order component had a strong positive correlation (0.616) with Vitamin A, but a strong negative correlation with protein (−0.910) and Zn (−0.772) regarding the DFP.

#### 3.3.4 The effects of second order component association on pod yield

The impact of the second-order component on pod yield is presented in Table 6. The results of the path analysis indicated that the NPP had the most positive direct effect on pod yield (0.784). The second-highest contributing attribute was APW (0.422), followed by PL (0.355) and the NSP (0.316), all of which had a positive and direct influence on pod yield. However, the traits such as D50%*F* (0.183) and DFP (0.293) showed a weaker relationship with pod yield. In contrast, plant height had a direct negative effect on pod yield (−0.324).

**Table 6:** Effect of second-order (yield components) components on pod yield.

Variable	D50%F	PH	NPP	NSP	APW	PL	DFP
D50%F	**0.183**	−0.261	0.208	0.192	0.008	0.003	−0.282
PH	0.389	**−0.324**	0.168	−0.283	0.005	−0.001	−0.228
NPP	0.128	−0.069	**0.784**	0.198	0.018	0.042	−0.109
NSP	0.276	−0.272	0.461	**0.316**	0.013	0.015	−0.186
APW	0.173	−0.078	0.642	0.196	**0.422**	0.037	−0.128
PL	0.027	0.009	0.592	0.091	0.015	**0.355**	−0.035
DFP	0.466	−0.252	0.292	0.214	0.010	0.007	**0.293**
Pod yield	0.352	−0.292	0.991***	0.644	0.863**	0.753*	0.444

Bold and diagonal lines represent direct effects. ***p < 0.001, **p < 0.01, *p < 0.05.

### 3.4 Principal component analysis for pod yield, yield components and nutritional components

**Figure 4:**
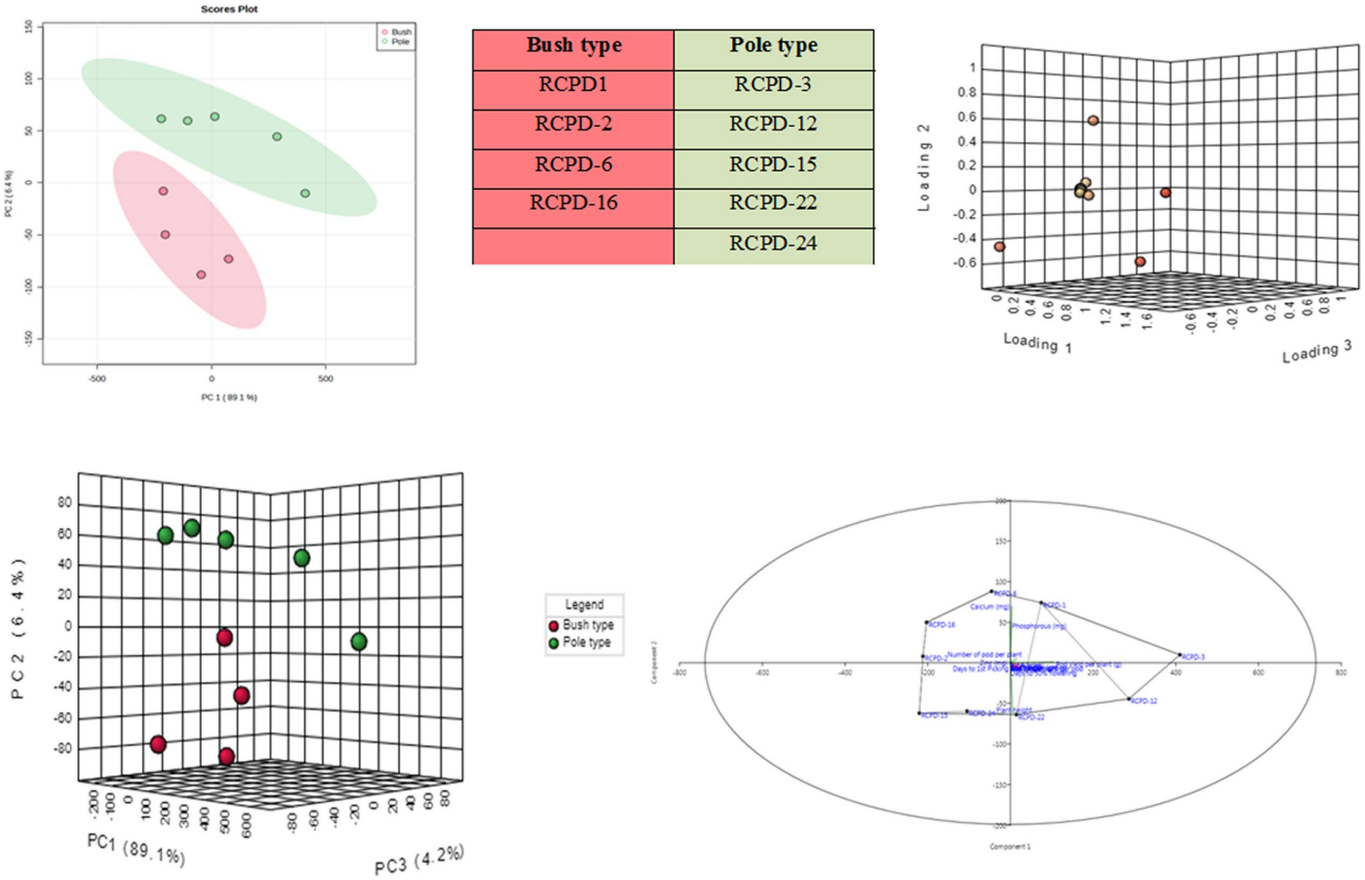
Represents the PCA (Principal component analysis); **(A)** two-dimensional and **(B)** three-dimensional PCA component loading plot for bush and pole type genotypes. The samples lying in each coordinate are clearly indicated; **(C)** indicated the three dimensional PCA score plot; **(D)** indicates two-dimensional component loading plot.

A principal component analysis (PCA) was conducted using all available lablab bean genotypes to assess the correlation between pod yield and nutritional quality traits, as well as to classify the genotypes based on these traits. By applying a minimum eigenvalue threshold of one, the original 14 traits were reduced to 8 principal components (PCs), which captured the entirety of the observed variation among the lablab bean genotypes (Table 7). The first principal component (PC1) explained 89.1% of the total variance in the dataset, while the second principal component (PC2) explained 6.4% of the variance (Figure 4). These two principal components were the main focus of interpretation as they collectively captured the majority of the variability in the data. PC1 was strongly and positively influenced by NPP, NSP, APW, and pod yield per plant. PC2 was strongly and positively influenced by PL, Ca, P, protein, and vitamin A.

**Table 7:** Eigenvectors from principal component (PC) analysis for the lablab bean genotypes.

Traits	PC 1	PC 2	PC 3	PC 4	PC 5	PC 6	PC 7	PC 8
Days to 50% flowering	0.009	−0.064	−0.027	0.337	−0.092	−0.532	0.077	0.188
Plant height	0.051	−0.595	−0.091	0.521	0.458	0.247	0.173	−0.073
Number of pod per plant	0.441	0.046	0.034	−0.034	0.380	0.198	−0.098	−0.071
Number of seed per pod	0.402	−0.008	−0.003	−0.014	0.012	0.021	−0.046	−0.063
Avg pod weight (g)	0.305	0.001	−0.007	0.005	−0.052	0.073	0.002	−0.089
Pod length (cm)	0.006	0.809	−0.004	−0.005	−0.025	0.219	0.336	0.006
Days to 1st picking	0.013	−0.061	−0.047	0.343	0.060	−0.560	−0.291	0.102
Protein (g)	−0.001	0.230	0.003	0.054	0.150	0.117	0.191	−0.058
Vitamin C (mg)	−0.002	−0.002	−0.008	0.089	−0.014	0.317	−0.190	0.178
Vitamin A (mcg)	0.014	0.280	0.012	0.540	−0.695	0.337	−0.106	−0.139
Zinc (mg)	−0.001	0.053	−0.001	−0.004	0.009	0.053	−0.011	−0.113
Iron (mg)	−0.002	−0.010	−0.004	−0.047	−0.012	−0.112	0.638	−0.384
Calcium (mg)	0.031	0.655	−0.627	0.290	0.241	0.068	0.099	−0.031
Phosphorous (mg)	−0.013	0.454	0.770	0.326	0.246	0.024	0.109	−0.015
Pod yield per plant (g)	0.988	0.011	0.031	−0.042	−0.072	−0.036	0.064	0.009
Eigenvalue	5.010	3.167	1.663	0.105	0.045	0.007	0.003	0.001
% variance	89.073	6.469	4.175	0.185	0.080	0.012	0.005	0.002
Cumulative	89.073	95.542	99.717	99.901	99.981	99.993	99.998	100.000

The aim of performing PCA was to visually represent the relationship between the lablab bean genotypes and their respective traits. This was accomplished by plotting PC1 (loading 1) against PC2 (loading 2) to differentiate the genotypes based on their traits. The lablab bean genotypes RCPD-1, RCPD-3, RCPD-12, and RCPD-22 were located on the positive side of PC1, indicating that these genotypes had high values for P, the NSP, and pod yield per plant. This suggests that these three criteria tend to vary together, such that when one increases, the others tend to increase as well. On the other hand, genotypes RCPD-6, RCPD-16, and RCPD-2 were located on the positive side of PC2, indicating that these genotypes had high values for the total NPP, Ca, and Zn.

The PCA distinguished different lablab bean genotypes with their specific trait association namely, (i) RCPD-6 is associated with calcium, RCPD-1 associated with P and (ii) RCPD-3 is associated with pod yield per plant, similarly RCPD-2 associated with NPP and Zn content.

### 3.5 Reaction of lablab bean genotypes for collar rot disease

Table 8 presented the results of the screening of lablab genotypes for resistance to collar rot in two consecutive years. The data showed that there were significant differences between the genotypes in terms of percent disease intensity. The wilt-susceptible check “Arka Sambhram” had the highest wilting percentages, at 57.2% during 2020–2021 and 55.6% during 2021–2022, among the genotypes screened, which confirmed the high and uniform infestation of collar rot in the soil of the sick plot and the effectiveness of the genotype screening process. During 2020–2021, the percent disease intensity among the genotypes ranged from 0 to 57.2%. Clear symptoms of pathogen attack on the collar zone of the host, followed by wilting and dieback, were first observed in the susceptible genotype 17 days after sowing. All test genotypes exhibited significantly lower disease intensity compared to the susceptible check “Arka Sambhram.” The line RCPD-15 recorded the maximum resistant reaction to collar rot as it exhibited the lowest rotting of 0% which was followed by the RCPD-24 (2.78%), RCPD-16 (2.78%), RCPD-1, and RCPD-22 (both 5.56%). Similarly, during 2020–2021, the percent disease intensity among the genotypes screened were ranged from 0 to 55.6%. The line RCPD-15 recorded the maximum resistant reaction to collar rot as it exhibited the no collar rot symptoms. The disease severity was severe in 2021–2022 as compared to 2020–2021. According to disease severity method, RCPD-15 was found to be resistant, RCPD-24, RCPD-16, RCPD-1, and RCPD-3 were moderately resistant, RCPD-22 and RCPD-2 were tolerant, RCPD-6 and RCPD-12 were moderately susceptible and Arka Sambharam was susceptible. It was interesting to notice that all dark purple color pod type genotypes were fall in resistant category.

**Table 8:** Reaction of lablab bean genotypes in two respective years and percentage of collar rot incidence during 2020–2021 to 2021–2022.

S.N	Genotype	Mean^(x)^ collar rot incidence (%)	
		2020–2021	2021–2022
1	RCPD-1	5.56(23.79)^a^	8.33(29.28)^b^
2	RCPD-2	11.11(33.98)^c^	13.89(38.19)^c^
3	RCPD-3	8.33(29.28)^b^	8.33(29.28)^b^
4	RCPD-6	25.00(52.36)^f^	25.00(52.36)^e^
5	RCPD-12	22.22(49.09)^e^	38.89(67.34)^f^
6	RCPD-15	0.00 (0.00)^a^	0.00(0.00)^a^
7	RCPD-16	2.78(16.74)^a^	5.56(23.79)^a^
8	RCPD-22	5.56(23.79)^a^	11.11(33.98)^b^
9	RCPD-24	2.78(16.74)^a^	2.78(16.74)^a^
	Arka Sambhram (Check)	57.22(75.76)^h^	55.56(84.11)^h^
C.D. at 5%		(4.61)	(5.62)

*Figures in parentheses are angular transformed values. “x” Means followed by the same letter in a column within each year are not significantly different (*p* ≤ 0.05) according to Fisher’s protected least significant difference test using values after angular transformation of the proportion of collar rot.

## 4 Discussion

Lablab bean is a highly adaptable crop that has immense potential for meeting subtropical and tropical food, animal feed, and soil enrichment concerns (2). The diversity richness provides high nutritional quality, making it an important underutilized crop with significant dietary benefits (28). In this study, results from the descriptive statistical analysis show substantial heterogeneity across the evaluated traits in lablab bean, as indicated by the high coefficient of variation values. This variation was particularly pronounced for vitamin A, which showed nearly five-fold differences between the genotypes. Similarly, there were two-fold differences in the contents of Fe and vitamin C. The study also identified that earliness is a desirable quality in lablab beans and the genotype RCPD-1 was found to be the earliest for 50% flowering, followed by RCPD-2 and RCPD-16. In the rice-fallow areas of South Asia, the drying of the top soil layer during harvest hampers the sowing of subsequent crops. To address this challenge, extra-early lablab genotypes can serve as valuable contributors for breeding early maturing varieties that align with the sowing windows of major crops. This adaptation enables the conversion of mono-cropped areas into double-cropped ones and the early maturity of lablab helps crops evade end-of-season stresses like drought and extreme temperatures (29–31).

Among the genotypes evaluated, RCPD-1 exhibited the shortest plant height, followed by RCPD-6 and RCPD-16. This indicates that these three genotypes possess a compact growth habit, which may have implications for their suitability for space requirements, mechanical harvesting and wind resistance (20, 31). In terms of pod yield, RCPD-3 RCPD-1 and RCPD-12 were found to have high yields due to more number of pods and high pod weight. The results revealed that the number of pods per plant and the average pod weight are the primary yield components that can be used as kick-starting points for selecting high-yielding genotypes. Furthermore, the alignment with earlier observations (15, 32) implies a certain level of reliability and generalizability of the findings.

### 4.1 Phenotypic correlation

Significant positive phenotypic correlations were observed between D50F with DFP, D50F with PH, PL with NPP, APW with NPP, protein with Zn, APW with Vit A, protein with Ca, and P, Vit C with Zn and NPP with pod yield which indicated that when selection is applied for one of these traits, an indirect improvement could be observed in the other traits (22). The results also indicated that selecting lablab genotypes for APW with NPP should have a positive influence on pod yield and should be considered when selecting for high yielding. Previous studies (32) in lablab bean showed similar trends. Earliness is a key trait for adaptation of lablab to short-season environments and to fit with the sowing windows of major crops. Traits related to earliness D50%F, DFP, and PH are highly correlated indicates that these traits are likely controlled by common underlying genetic factors. Breeders can leverage this information to select for desired trait combinations and potentially improve correlated traits simultaneously (14, 20). Significant positive phenotypic correlations were observed among most of the measured nutritional components, which indicated that these traits could be improved and selected together. The corroborating evidence from previous studies also implies a level of continuity in the understanding of the traits and characteristics being investigated (14, 15).

The study found that there were strong positive correlations between protein, Zn, Can, and P in lablab bean. This suggests that selecting for protein content could lead to enhanced nutritional value in lablab bean. However, measuring all of the nutritional components can be costly for smaller breeding programs. Therefore, the option of indirectly selecting for most of the nutritional components in this bean through selecting for protein would be highly beneficial for breeding programs with limited resources. Zn was negatively correlated with D50F, DFP, and PH indicating early genotypes may has raised level of zinc as compared to late genotypes. There was no notable correlation between Fe and any of the studied traits. This suggests that Fe can be enhanced without relying on the improvement of any of the traits. Similar results were reported by Mbuma et al. (14) in cowpea.

### 4.2 Genotypic correlations

Genetic correlation is a technique utilized to assess the genetic association between two distinct traits within a population, commonly referred to as the correlation of breeding values (33). By evaluating the additive genetic variance to ascertain the breeding values, this method helps in determining the inheritance of a particular trait. Hence, genetic correlation holds significant importance for plant breeders. The analysis of genetic correlation showed that NPP has a strong and statistically significant correlation with PL and APW. Selecting plants for higher NPP indirectly leads to selection of traits related to PL and APW, showing additive genetic effects and positive correlations with pod yield. A multi-trait selection approach multiplying the impact of the effort and enhances overall crop productivity. A similar correlation trend was found in common bean (19), lablab bean (17), garden pea (20), cowpea (34), and bambara groundnut (18). The study also found a notable and positive correlation between NPP and P, suggesting that agro-morphological and nutritional traits could potentially be improved simultaneously. The protein significantly positively correlated with Zn indicating that an improvement of the protein contents would have a positive influence on zinc content. The results also indicated possible simultaneous improvement of zinc and protein contents, indicating the potential of lablab bean toward mitigating protein malnutrition, nutritional deficiency and providing health benefits.

### 4.3 Path coefficient analysis

Path coefficient analysis is a useful method for examining both direct and indirect correlations between component characteristics by partitioning correlation coefficients. By breaking down the correlation coefficients into path coefficients, researchers can gain a more detailed understanding of the underlying mechanisms that drive these relationships. Path coefficient analysis has been studied in several crops including lablab bean (35, 36) bambara groundnut (18), and cowpea (34). In our study, path coefficient analysis was conducted for pod yield against yield components separately and pod yield against yield components and nutritional quality traits combinedly. According to our study, the most influential factor on pod yield per plant was the average weight of the pods, followed by number of pods per plant and protein content. Although APW and NPP had the most significant direct effects on pod yield, the number of pods per plant, average weight of pods, and pod length had the highest indirect effects on pod yield. Therefore, a comprehensive approach that considers both direct and indirect effects is crucial for optimizing pod yield in lablab bean. Selecting for these traits directly would lead to simultaneous improvement in these traits and overall yield (36).

The first-order component, which consists of nutritional parameters, was found to have a significant impact on the second-order components. Specifically, the analysis revealed that vitamin A had a strong positive association with the days to 50% flowering and plant height, while protein and calcium had a strong negative correlation with these parameters. Moreover, there was a significant positive correlation between the number of pods per plant and both protein and vitamin A content. Similarly, average pod weight showed the highest positive correlation with vitamin A content, followed by zinc and protein content. These findings highlight vitamin A has positive association with all yield related traits and possibility of simultaneous improvement of pod yield and vitamin A (14). The results also indicated possible simultaneous improvement of vitamin A and yield components.

### 4.4 Principal component analysis

PCA is a statistical method, enables us to observe the associations between different traits and the trait profile of genotypes in a clear and concise graphical representation (37). The first principal component (PC1) represents the direction of maximum variability in the data, and the second principal component (PC2) represents the direction of maximum variability that is orthogonal to PC1. By plotting PC1 against PC2, it is possible to differentiate between different genotypes based on their associated traits. For interpretation of data, biplot or principal component analysis (PCA) is an important and useful statistical program (38, 39). The analysis showed that genotypes RCPD-1, RCPD-3, RCPD-12, and RCPD-22 had high levels of phosphorus, number of seeds per pod, and pod yield per plant, and were located on the positive side of PC1. This suggests that these three components are positively correlated and tend to vary together. Similarly, genotypes RCPD-6, RCPD-16, and RCPD-2 were displayed on the positive side of PC2, indicating that these genotypes had high values for total number of pods per plant, calcium, and zinc. This suggests that these three criteria are positively correlated and tend to vary together (17). The considerable diversity observed among these lablab bean genotypes in terms of pod yield and nutritional quality traits suggests that they hold great potential for use in breeding programs and for developing new cross combinations (22, 38). This can help to expand the genetic diversity of lablab and ultimately lead to the creation of high-yielding genotypes with superior nutritional value.

### 4.5 Collar rot disease reaction

The fungus *S. rolfsii*, which causes collar rot, is prevalent in tropical and subtropical regions as well as the warmer regions of the temperate zone across the globe (23). However, with the current climate change situation, the disease has become more severe due to the increased conducive temperature and humidity levels, leading to significant economic losses in lablab bean (40, 41). The most effective and cost-efficient approach to preventing collar rot occurrences in lablab cultivation is by cultivating disease-resistant varieties of lablab bean. In present study, resistant (RCPD-15) and moderately resistant genotypes (RCPD-24, RCPD-16, RCPD-1, and RCPD-3) were identified. The diverse reactions observed in different genotypes could be attributed to their varying genetic backgrounds. Therefore, further investigation is required to conduct detailed genetic studies on these genotypes, particularly in relation to their resistance to collar rot.

## 5 Conclusion

Multiple trait selection may be possible due to the significant positive genetic and phenotypic correlations between traits. The study supported that enhancing certain yield related traits can lead to an improvement in some nutritional traits of lablab bean as yield related traits are easier to measure than nutritional traits. Specifically, a notable positive indirect effect was observed between NPP and P, protein, and Zn. Additionally, APW and NPP are crucial attributes among the tested yield components due to their strong positive direct effect on pod yield. Therefore, to optimize lablab bean production and enhance its nutritional value, it is essential to consider these components. The PCA identified RCPD-1, RCPD-3, RCPD-12, and RCPD-22 had high values for phosphorus, number of seed per plant (NPP), and pod yield per plant and RCPD-6, RCPD-16, and RCPD-2 had high values for NPP, calcium, and zinc. The outcomes of this study will be utilized as selection standards for improving lablab bean production and simultaneously for the biofortification program. Furthermore, the findings will suggest that the resistant genotypes (RCPD-15, RCPD-24, RCPD-16, RCPD-1, and RCPD-3) identified in this study possess high levels of resistance to collar rot disease resistance and can be used as donor in lablab bean improvement within the India and other parts of the world.

## Data availability statement

The original contributions presented in the study are included in the article/supplementary material, further inquiries can be directed to the corresponding author.

## Author contributions

KS and AC: design the research, perform the experiments, and writing–original draft. AKD: disease scoring, KT, RK, STK, and AM: data analysis, review, and editing. JL, SK, UK, and AD: project administration, resources, and supervision. All authors have read, revised, and approved the manuscript.

## Conflict of interest

The authors declare that the research was conducted in the absence of any commercial or financial relationships that could be construed as a potential conflict of interest.

The reviewer PC declared a shared parent affiliation with the authors to the handling editor at the time of review.

## Publisher’s note

All claims expressed in this article are solely those of the authors and do not necessarily represent those of their affiliated organizations, or those of the publisher, the editors and the reviewers. Any product that may be evaluated in this article, or claim that may be made by its manufacturer, is not guaranteed or endorsed by the publisher.
