# Supplementary material for: Evaluation of lablab bean [Lablab purpureus (L.) sweet] genotypes: unveiling superior pod yield, nutritional quality, and collar rot resistance
Source: Front Nutr. 2024 Jan 11;10:1243923. doi: 10.3389/fnut.2023.1243923 (PMC10809392; doi:10.3389/fnut.2023.1243923)
Supplement: Supplementary file 1 [file Data_Sheet_1.docx]

## Supp. Table S1 Description of experimental materials used for nutritional analysis

| **Genotype** | **Plant growth** | **Pigmentation on stem** | **Leaf colour** | **Flower colour** | **Pigmentation on pod** | **Photo-sensitivity** | **Thermo-sensitivity** |
| --- | --- | --- | --- | --- | --- | --- | --- |
| RCPD-1  (IC-645155) | Determinate | Absent | Green | White | Absent | Photo-insensitive | Thermo-sensitive |
| RCPD-2 | Semi-determinate | Absent | Green | White | Absent | Photo-insensitive | Thermo-sensitive |
| RCPD-3  (IC-645156) | Indeterminate | Absent | Green | Light purple | Absent | Photo-insensitive | Thermo-sensitive |
| RCPD-6 | Determinate | Absent | Green | White | Absent | Photo-insensitive | Thermo-sensitive |
| RCPD-12 | Indeterminate | Present | Dark green with purple midrib and vein | Purple | Present | Photo-insensitive | Thermo-sensitive |
| RCPD-15 | Indeterminate | Absent | Light green | White | Absent | Photo-insensitive | Thermo-sensitive |
| RCPD-16 | Determinate | Absent | Green | White | Absent | Photo-insensitive | Thermo-sensitive |
| RCPD-22 | Indeterminate | Absent | Dark green | Purple | Absent | Photo-insensitive | Thermo-sensitive |
| RCPD-24 | Indeterminate | Present | Dark green with purple midrib and vein | Purple | Present | Photo-insensitive | Thermo-sensitive |

| **S.N** | **Agronomic Practices** | ***Rabi* 2020-21** | ***Rabi* 2021-22** | **Pre *Kharif** 2021-22** |
| --- | --- | --- | --- | --- |
| 1 | Sowing time | 1^st^ week of October | 1^st^ week of October | 1^st^ week January |
| 2 | Spacing (P×R) in meter | 1×1 | 1×1 | 1×1 in ridge bed |
| 3 | Fertilization | FYM: 12.5, Nitrogen: 25kg/ha  Phosphorous: 50kg/ha | FYM: 12.5, Nitrogen: 25kg/ha  Phosphorous: 50kg/ha | FYM: 12.5, Nitrogen: 25kg/ha  Phosphorous: 50kg/ha |
| 4 | After cultivation (for pole type genotypes) | Provide stakes to reach pandal of 5 m height and train the vines on pandal | Provide stakes to reach pandal of 5 m height and train the vines on pandal | Provide stakes to reach pandal of 5 m height and train the vines on pandal |
| 5 | Irrigation once in a week | Once in a week | Once in a week | Once in a week |
| 6 | Fungicide | No fungicide was applied to screen collar rot resistance lines | No fungicide was applied to screen collar rot resistance lines | No fungicide was applied to screen collar rot resistance lines |
| 7 | Insecticide | - | - | Applied to check whitefly population |

**Supp. Table S2 Standard cultivation technique followed to grow lablab bean at ICAR-RCER, Patna, Bihar, India**

**Two evaluate photosensitivity and thermo sensitivity grown twice season in 2021-22*

**Supp. Table S3 Description of agronomic traits of twenty five genotype used in this study**

| **S.N** | **Genotypes** | **Days to 50% flowering** | **Plant height (cm)** | **No. of pod per plant** | **No. of seed per pod** | **Avg pod weight (g)** | **Pod length (cm)** | **Days to 1st Picking** | **Pod yield per plant (g)** |
| --- | --- | --- | --- | --- | --- | --- | --- | --- | --- |
| **1** | RCPD-1 | 40 | 60 | 110 | 5 | 7.96 | 12.33 | 57 | 870 |
| **2** | RCPD-2 | 45 | 90.33 | 58 | 5 | 5.9 | 12 | 60 | 384.33 |
| **3** | RCPD-3 | 57 | 127.67 | 133 | 6 | 9.23 | 13.67 | 75 | 997 |
| **4** | RCPD-4 | 58 | 130 | 45 | 4 | 5.4 | 13 | 75 | 240 |
| **5** | RCPD-5 | 62 | 125 | 57 | 5 | 9.2 | 16 | 78 | 520 |
| **6** | RCPD-6 | 52 | 66.67 | 88 | 4 | 5.7 | 11.67 | 69 | 548.33 |
| **7** | RCPD-7 | 56 | 135 | 35 | 4 | 8.2 | 12 | 71 | 268 |
| **8** | RCPD-8 | 53 | 180 | 42 | 5 | 4.3 | 14 | 72 | 180 |
| **9** | RCPD-9 | 53 | 70 | 58 | 6 | 5.9 | 16 | 69 | 267 |
| **10** | RCPD-10 | 60 | 103 | 67 | 5 | 8.41 | 13 | 72 | 601 |
| **11** | RCPD-11 | 57 | 150 | 45 | 4 | 9.9 | 12 | 75 | 439 |
| **12** | RCPD-12 | 53 | 154 | 134 | 6 | 6.7 | 13.13 | 73 | 875 |
| **13** | RCPD-13 | 62 | 156 | 54 | 5 | 4.8 | 12.5 | 80 | 248 |
| **14** | RCPD-14 | 60 | 135 | 80 | 6 | 5.2 | 10 | 78 | 408 |
| **15** | RCPD-15 | 55 | 147.33 | 59 | 5 | 5.57 | 9.47 | 73 | 973 |
| **16** | RCPD-16 | 44 | 67.67 | 65 | 5 | 7.67 | 9.13 | 63 | 792.33 |
| **17** | RCPD-17 | 60 | 150 | 57 | 6 | 9.6 | 11.5 | 74 | 537 |
| **18** | RCPD-18 | 57 | 144 | 69 | 5 | 9.1 | 10.6 | 72 | 623 |
| **19** | RCPD-19 | 54 | 75 | 94 | 4 | 8.5 | 8 | 69 | 750 |
| **20** | RCPD-20 | 60 | 150 | 67 | 6 | 5.1 | 10.5 | 76 | 321 |
| **21** | RCPD-21 | 62 | 135 | 76 | 3 | 4.7 | 11.2 | 74 | 348 |
| **22** | RCPD-22 | 57 | 142.67 | 89 | 5 | 6.67 | 11.33 | 72 | 608.33 |
| **23** | RCPD-23 | 56 | 145 | 79 | 4 | 9.8 | 9.2 | 78 | 771 |
| **24** | RCPD-24 | 53 | 134.67 | 75 | 5 | 6.63 | 8 | 68 | 490.67 |
| **25** | Arka Sambhram | 50 | 50 | 62 | 4 | 4.1 | 10.2 | 72 | 254.2 |

**Supp. Table S4 Standard rating scale of resistance and susceptibility of plants developed by ICRISAT**

| **Scale** | **Mortality** | **Reaction** |
| --- | --- | --- |
| 1 | 0 | Resistant (R) |
| 2 - 3 | 10 or less | Moderately Resistant (MR) |
| 4 - 5 | 11-20 | Tolerant (T) |
| 6 - 7 | 21-50 | Moderately Susceptible (MS) |
| 8 - 9 | 51- above | Susceptible (S) |
